# Jeopardizing quality at the frontline of healthcare: prevalence and risk factors for disrespect and abuse during facility-based childbirth in Ethiopia

**DOI:** 10.1093/heapol/czx180

**Published:** 2017-12-22

**Authors:** Kathleen P Banks, Ali M Karim, Hannah L Ratcliffe, Wuleta Betemariam, Ana Langer

**Affiliations:** 1 Department of Global Health and Population, Women and Health Initiative, Harvard T.H. Chan School of Public Health, 651 Huntington Avenue, FXB 7th Floor, Boston, MA 02115, USA,; 2 Department of Global Health, Boston University School of Public Health, 801 Massachusetts Ave, Crosstown Building, 3rd Floor, Boston, MA 02118, USA,; 3 JSI Research & Training Institute Inc., The Last Ten Kilometers Project, 44 Farnsworth St, Boston, MA 02210, USA, and; 4 Ariadne Labs at Brigham and Women’s Hospital and the Harvard T.H. Chan School of Public Health, 401 Park Drive, Boston, MA 02215, USA

**Keywords:** Quality of care, maternal health, disrespect and abuse, primary health care, maternity services

## Abstract

Disrespect and abuse (D&A) experienced by women during facility-based childbirth has gained global recognition as a threat to eliminating preventable maternal mortality and morbidity. This study explored the frequency and associated factors of D&A in four rural health centres in Ethiopia. Experiences of women who delivered in these facilities were captured by direct observation of client-provider interaction (*N* = 193) and exit interview at time of discharge (*N* = 204). Incidence of D&A was observed in each facility, with failure to ask woman for preferred birth position most commonly observed [*n* = 162, 83.9%, 95% confidence interval (95% CI) 78.0–88.5%]. During exit interviews, 21.1% (*n* = 43, 95% CI 15.4–26.7%) of respondents reported at least one occurrence of D&A. Bivariate models using client characteristics and index birth experience showed that women’s reporting of D&A was significantly associated with childbirth complications [odds ratio (OR) = 7.98, 95% CI 3.70, 17.22], weekend delivery (OR = 0.17, 95% CI 0.05, 0.63) and no previous delivery at the facility (OR = 3.20, 95% CI 1.27, 8.05). Facility-level fixed-effect models found that experience of complications (OR = 15.51, 95% CI 4.38, 54.94) and weekend delivery (OR = 0.05, 95% CI 0.01–0.32) remained significantly and most strongly associated with self-reported D&A. These data suggest that addressing D&A in health centres in Ethiopia will require a sustained effort to improve infrastructure, support the health workforce in rural settings, enforce professional standards and target interventions to improve women’s experiences as part of quality of care initiatives.

**Key Messages**

- Disrespect and abuse (D&A) experienced by women during facility-based childbirth has gained global recognition as a threat to eliminating preventable maternal mortality and morbidity.
- This study sought to quantify the frequency and categories of D&A experienced by women in four health centres in two rural regions of Ethiopia for the purposes of developing a community-led intervention. Experiences of women who delivered in these facilities were captured by direct observation of client-provider interaction and exit interview at time of discharge.
- During exit interviews, 21.1% of respondents reported at least one occurrence of D&A. Failure to ask woman for preferred birth position most commonly observed during client-provider interactions (83.9%). Complications during childbirth and time of delivery were significantly associated with reported D&A.
- Addressing D&A in health centres in Ethiopia will require a shift in priorities towards improving the experience and quality of care, a sustained effort to improve health care centres’ infrastructure and greater support of the rural health workforce.

## Introduction

Complications from pregnancy and childbirth are the leading causes of maternal mortality and morbidity for women of reproductive age in developing countries (Kassebaum *et al.* 2014). It is widely acknowledged that maternal deaths can be prevented if women have access to skilled childbirth services within a formal health care system (Campbell *et al.* 2016). However, evidence has shown that access to maternal health services alone is insufficient to prevent maternal deaths, and that the quality of care received is integral to ensuring good maternal health outcomes (Souza *et al.* 2013). This is reflected in the post-2015 development agenda, which underlines the growing recognition of the importance of quality in health service delivery (Koblinsky *et al.* 2016). The WHO Quality of Care framework for maternal and newborn health expanded the definition of ‘quality of care’ to give equal value to clinical (or ‘technical’) quality and experiences of care, while the Strategies towards Ending Preventable Maternal Mortality (EPMM) working group highlighted priority recommendations for eliminating discrimination and developing health systems that can deliver interventions both effectively and equitably (Tunçalp *et al.* 2015; World Health Organization 2015).

Disrespect and abuse (D&A) experienced by women during facility-based childbirth is gaining recognition as violation of women’s rights (Ogangah *et al.* 2007; Freedman *et al.* 2014; Sando *et al.* 2014; Abuya et al. 2015). Further, D&A has been acknowledged as a deficiency in the delivery of high quality maternal health services, threatening the ability of health systems to achieve good maternal health outcomes (Bowser and Hill 2010; Kruk *et al.* 2014; Abuya et al. 2015a; Bohren *et al.* 2015; Vogel *et al.* 2015a,b; Sando *et al.* 2016). D&A manifests as physical violence, harsh language, stigma and neglect suffered by women at the hands of health care providers (Bowser and Hill 2010; Kruk *et al.* 2014; Abuya et al. 2015a; Bohren *et al.* 2015; Ratcliffe et al. 2016a). Drivers of D&A can include systemic failures, such as overwhelmed health care administration, poor staffing and supervisory structures and inadequate physical infrastructure (Bowser and Hill 2010; Freedman and Kruk 2014; Ratcliffe *et al.* 2016b). Women who experience D&A are more likely to report lower satisfaction with their birth experience and are less likely to seek facility-based delivery for future pregnancies (Kujawski *et al.* 2015).

Such considerations are important for Ethiopia, which, with 353 maternal deaths per 100 000 live births, has one of the highest maternal mortality ratios in the world (WHO, UNICEF, UNFPA 2015). The Ethiopian Federal Ministry of Health has worked to improve access to facility-based maternal health services by dramatically increasing the number of primary health centres (‘health centres’) and eliminating user fees, yet the national facility delivery rate in Ethiopia remains low at 15% (Central Statistical Agency 2014). Ethiopian women who have access to health facilities often choose to give birth elsewhere, even among women for whom the benefits of facility-based childbirth have been demonstrated (Kruk *et al.* 2010). Poor provider attitudes—including harassment, lack of attention to complaints, and lack of follow-up in labour—have been cited as deterrents to the use of facility services (Belay *et al.* 2007). Additional studies in Ethiopia have found that women perceive health care providers to be insensitive and unduly harsh (Berhane *et al.* 2001) and unresponsive to community beliefs and practices. Thus, women’s poor experiences with care at health centres may be deterring them from seeking childbirth services, undermining existing national efforts to prevent maternal mortality.

Colleagues have investigated respect and dignity during perinatal care in tertiary hospitals in Ethiopia, (Asefa and Bekele 2015; Rosen *et al.* 2015). However, to our knowledge, the prevalence of D&A reported by women at health centres in rural Ethiopia, the level of the health system at which women are encouraged to seek facility-based delivery, had never been investigated. This study sought to quantify the frequency and categories of D&A experienced by women in four health centres in two rural regions of Ethiopia for the purposes of developing a community-led intervention. As part of this endeavour, we identified factors associated with reporting D&A to identify the most appropriate area for intervention. Understanding the factors associated with D&A will assist in local and national efforts to improve the quality of care, increase rates of facility-based delivery and improve maternal health outcomes in a primary health care setting.

## Methods

### Study design

The study was a cross-sectional design to assess manifestations of D&A among women who gave birth in four rural health centres in Amhara and Southern Nations, Nationalities, and Peoples (SNNP) regions of Ethiopia during July–September 2013. Client-provider interactions during labour and delivery were observed for 193 births, and 204 women who gave birth at these health centres were interviewed at their time of discharge from the facility (*n* = 204). Health workers in the participating health centres were aware that the quality of client-provider interactions was being captured through direct observation and women surveyed.

### Study setting

To ensure universal coverage of primary health care, the Government of Ethiopia has been investing substantially to develop the district- or *woreda*^1^-level health system, which encompasses a primary hospital with four to five primary health care units (PHCUs). Each PHCU is comprised of one health centre that serves a population of approximately 25 000, and five satellite health posts. Designed to be the front line of service delivery for childbirth, the health centres are staffed with health officers, nurses and midwives to provide primarily curative care, including basic emergency obstetric and newborn care (BEmONC). Additionally, the health centres receive referrals from the health posts, and provide essential supplies, technical and administrative support to the health posts.^2^

The study was conducted in four health centres that are supported by the Last Ten Kilometers (L10K), a technical support program for the Ethiopian Federal Ministry of Health that aims to strengthen the links between households, communities and the formalized health care system. Two of the study health centres were in Amhara region and two were in Southern Nations, Nationalities and Peoples’ region (SNNP). Amhara (population 20.3 million) and SNNP (population 18.9 million) are respectively the second and third most populous regions in Ethiopia, representing about 46% of the population of the country. The four health centres were purposively selected among fourteen health centres that were part of the L10K’s ongoing clinical quality improvement activities. To account for any differences in D&A associated with patient flow, the four health centres were sampled such that each region included one high volume health centre (>450 deliveries annually) and one low volume health centre (fewer than 150 deliveries annually). At each health centre, maternity staff provided care in three units during the weekdays (family planning, antenatal care, and labour and delivery), while only labour and delivery services were provided at night and during the weekends.

### Data collection

Study instruments were adapted from the tools developed by the Population Council in Kenya to fit the Ethiopian context (Abuya et al. 2015a). The client-provider interaction tool was administered in English, the ‘language of medicine’ in Ethiopia. The exit interview tool was administered in Amharic; the original English tool was translated into Amharic and then translated back to English to ensure consistency.

Twelve data collectors and four supervisors with bachelor’s degrees in health sciences who were working concurrently as healthcare providers in non-study health facilities were responsible for data collection. One full day of training was provided to data collectors and supervisors to familiarize them with the instrument and methods for interview techniques for sensitive material, including appropriate recording, acquiring informed consent and ensuring confidentiality. Data collectors and supervisors were trained to administer both the client-provider interaction and women’s exit interview tools. Three data collectors and one supervisor were deployed to each health centre. Data collectors worked in 8-h shifts to allow for continuous coverage at the health centre.

All women who gave birth in the four health centres during the study period were eligible to participate in the study; there were no refusals. The expected sample size of 246 was based on an assumed prevalence of D&A of 20% across all study sites assuming ±5% precision and 95% confidence interval (95% CI), an estimate based on a similar study in Kenya (Warren et al. 2013).

Client-provider interactions were captured through one-to-one direct observation from a woman’s time of entry at the health centre, throughout the labour and delivery period, until her time of entry to the post-natal ward. The data collector used a structured observation tool to capture specific manifestations of D&A. If the observation period continued beyond the 8-h shift, the data collector handed over the observation tool to the arriving data collector. The exit interview was conducted at the time of discharge, approximately three to 6 h post-partum; the questionnaire focused on the woman’s perceived experiences of D&A during her labour and delivery at the health centre.

Written consent was obtained from women during the first stage of labour at admittance. All records and data collection tools were assigned an anonymous code, and identifiers were not used in the analysis or final reporting. Ethical clearance was obtained from Institutional Review Boards at the Amhara and SNNP Regional Health Bureaus and an ethical clearance waiver for secondary data analysis was obtained from the Harvard T.H. Chan School of Public Health Institutional Review Board.

### Measurements

**Figure 1.**
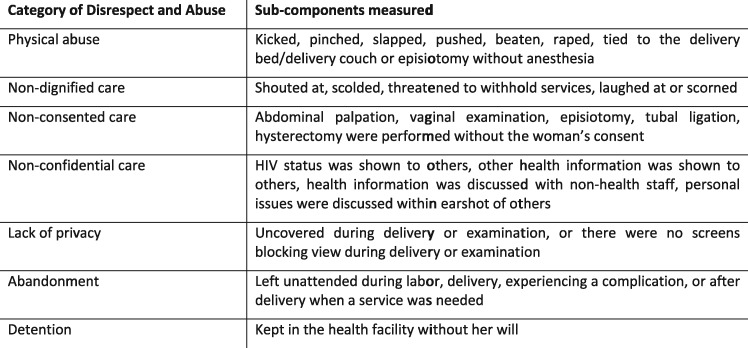
Categories of disrespect and abuse.

D&A were operationalized using an adaptation of the seven categories described in Bowser and Hill’s landscape analysis (Bowser and Hill 2010) (see Figure 1). Indicators for observed events of D&A were identified through the literature and local BEmONC protocol. Since observing D&A is inherently subjective and heavily based on local norms (Freedman *et al.* 2014), each item on the client-provider interaction tool was reviewed during data collection training to determine local consensus on the manifestation of D&A and practiced among data collectors to ensure consistency in recording. As part of a larger global consensus on describing and defining prevalence from the perspective and experience of the woman (Freedman *et al.* 2014; Kruk *et al.* 2014; Abuya et al. 2015a; Sando *et al.* 2016), prevalence of each of D&A category was calculated using the exit interview data. Women who reported experiencing one or more sub-components of D&A were included in the overall prevalence measure.

**Figure 2.**
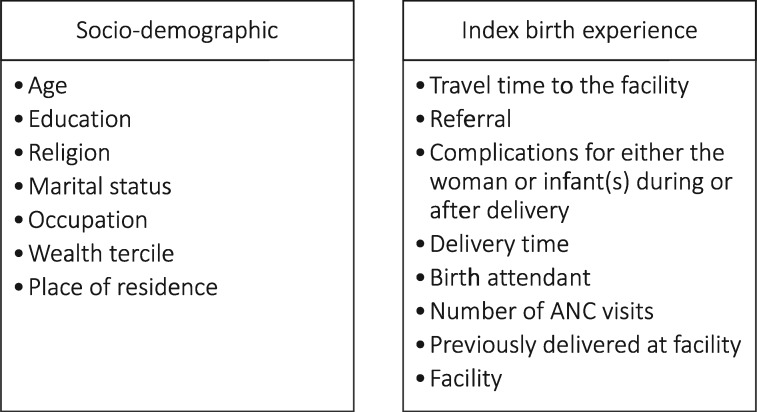
Independent variables.

Independent variables were chosen based on hypotheses that women from certain sub-groups, previous history with the healthcare system and/or individual experiences with the index birth, including birth complications,^3^ may be more likely to report D&A (see Figure 2).

### Data management and analysis

Study instruments were thoroughly examined for completion and accuracy by the data supervisors. Ten percent of the data entries were randomly selected and checked for consistency; frequencies were used to check for outliers and cleaning of data.

Differentials in socio-demographic and index birth variables of women in the samples of the four health centres were assessed using bivariate analyses. The prevalence of observed and reported D&A was estimated and whether it varied by facility were assessed using Fisher’s exact test. Vast variations between observed and reported D&A were expected due to cultural norms, varying perspectives on quality of care, and previous experience using the tools in a related study in Dar es Salaam, Tanzania (Sando *et al.* 2016).

Then, bivariate and multivariate analyses were performed to examine the unadjusted and adjusted relationships between selected socio-demographic and index birth experience. Fisher’s exact test was used for the bivariate analysis and facility-level fixed effects logistic regression was used to estimate adjusted relationships between D&A and the independent variables. Statistical significance was considered at two-tailed *P*-value <0.05. Stata 14.0 was used for the analysis (StataCorp 2015).

## Results

### Demographic and index birth characteristics

The demographic characteristics and birth experiences of respondents from each health centre are shown in Table 1. Overall, client characteristics varied significantly between health centres. More women from the SNNP region were from a rural *kebele* (village) than those in the Amhara region (*P* < 0.001). Religious affiliation differed substantially between the study sites as well (*P* < 0.001), with all respondents who self-identified as Muslim concentrated in the SNNP region’s high volume (SHV) health centre. Wealth tercile^4^ distribution varied significantly between health centres (*P* < 0.001), with women from the SNNP region low volume health centre (SLV) and the Amhara region high volume health centre (AHV) tending to be poorer than women from the Amhara region low volume health centre (ALV) and SHV. Occupation (*P* < 0.001) was significantly differentially distributed by health centre; 71% of women from ALV identified as farmers and 71% of SLV identified as homemaker. Educational attainment was significantly different across sites (*P* < 0.05), with women from low volume health centres less likely to have formal education than women from high-volume sites.

**Table 1.** Background characteristics and birth experience of exit interview respondents in four facilities in Amhara & SNNP region, Ethiopia, 2013

Characteristics	Total (*N* = 204)	Amhara		SNNP		*P*-value
		High volume (*N* = 77)	Low volume (*N* = 21)	High volume (*N* = 71)	Low volume (*N* = 35)	
	*n* (%)	*n* (%)	*n* (%)	*n* (%)	*n* (%)	
Age group						
16–24	73 (35.8)	29 (37.7)	7 (33.3)	20 (28.2)	17 (48.6)	0.180
25–34	106 (52.0)	35 (45.5)	11 (52.4)	43 (60.6)	17 (48.6)	
35–45	25 (12.3)	13 (16.9)	3 (14.3)	8 (11.3)	1 (2.9)	
Education						
None	89 (43.6)	35 (45.5)	11 (52.4)	37 (52.1)	6 (17.1)	0.023***
Primary	73 (35.8)	25 (32.5)	7 (33.3)	21 (29.6)	20 (57.1)	
Secondary+	42 (20.6)	17 (22.1)	3 (14.3)	13 (18.3)	9 (25.7)	
Religion						
Christian	137 (67.2)	77 (100.0)	21 (100.0)	4 (5.6)	35 (100.0)	<0.001*
Muslim	67 (32.8)	0 (0.0)	0 (0.0)	67 (94.4)	0 (0.0)	
Marital status						
Other	14 (6.9)	6 (7.8)	2 (9.5)	4 (5.6)	2 (5.7)	0.871
Married	190 (93.1)	71 (92.2)	19 (90.5)	67 (94.4)	33 (94.3)	
Occupation						
Homemaker	78 (38.2)	26 (33.8)	3 (14.3)	24 (33.8)	25 (71.4)	<0.001*
Farming	71 (34.8)	30 (39.0)	15 (71.4)	22 (31.0)	4 (11.4)	
Other	55 (27.0)	21 (27.3)	3 (14.3)	25 (35.2)	6 (17.1)	
Wealth tercile						
Poorest	68 (33.3)	26 (33.8)	5 (23.8)	20 (28.2)	17 (48.6)	<0.001*
Medium	68 (33.3)	30 (39.0)	9 (42.9)	14 (19.7)	15 (42.9)	
Least poor	68 (33.3)	21 (27.3)	7 (33.3)	37 (52.1)	3 (8.6)	
Place of residence						
Rural	130 (63.7)	36 (46.8)	13 (61.9)	50 (70.4)	31 (88.6)	<0.001*
Urban	74 (36.3)	41 (53.3)	8 (38.1)	21 (29.6)	4 (11.4)	
Travel time to reach the facility						
<1 h	123 (60.3)	39 (50.7)	8 (38.1)	50 (70.4)	26 (74.3)	0.002**
1–2 h	52 (25.5)	25 (32.5)	7 (33.3)	17 (23.9)	3 (8.6)	
>2 h	29 (14.2)	13 (16.9)	6 (28.6)	4 (5.6)	6 (17.1)	
Referred to the facility						
Referred	10 (4.9)	3 (3.9)	3 (14.3)	2 (2.8)	2 (5.7)	0.194
Came directly	194 (95.1)	74 (96.1)	18 (85.7)	69 (97.2)	33 (94.3)	
Any complications						
No	129 (63.2)	45 (58.4)	9 (42.9)	46 (64.8)	29 (82.9)	0.014***
Yes	75 (36.8)	32 (41.6)	12 (57.1)	25 (35.2)	6 (17.1)	
Delivery time						
Weekdays day	91 (44.6)	38 (49.4)	8 (38.1)	30 (42.3)	15 (42.9)	0.883
Weekdays night	65 (31.9)	25 (32.5)	6 (28.6)	23 (32.4)	11 (31.4)	
Weekend	48 (23.5)	14 (18.2)	7 (33.3)	18 (25.4)	9 (25.7)	
Birth attendant						
Health officer	51 (25.0)	8 (10.4)	12 (57.1)	11 (15.5)	20 (57.1)	<0.001*
Nurse	36 (17.7)	24 (31.2)	0 (0.0)	1 (1.4)	11 (31.4)	
Midwife	117 (57.4)	45 (58.4)	9 (42.9)	59 (83.1)	4 (11.4)	
Number of ANC visits						
0–2	29 (14.2)	13 (16.9)	6 (28.6)	5 (7.0)	5 (14.3)	0.062****
3–4	175 (85.8)	64 (83.1)	15 (71.4)	66 (93.0)	30 (85.7)	
Previously delivered at the facility						
Yes	61 (29.9)	14 (18.2)	3 (14.3)	34 (47.9)	10 (28.6)	<0.001*
No	143 (70.1)	63 (81.8)	18 (85.7)	37 (52.1)	25 (71.4)	
Previously used the facility						
Yes	154 (75.5)	56 (72.7)	13 (61.9)	65 (91.6)	20 (57.1)	<0.001*
No	50 (24.5)	21 (27.3)	8 (38.1)	6 (8.5)	15 (42.9)	

* *P* < 0.001;

** *P* < 0.01; ***P < 0.05; ****P < 0.1.

Nearly all respondents (95.1%) reported coming directly to the health centre for delivery without a referral. For most respondents, travel to the health centre took less than 1 h, though this varied significantly by health centre (*P* < 0.005) and women from ALV reported longer travel times overall. More than 85% of respondents completed at least three or four antenatal care visits. Approximately one-third of respondents reported that they or their infant experienced some type of complication; women from SNNP health centres were less likely to report any form of complication. Nearly half of deliveries occurred during the day on weekdays, with no significant difference between health centre, and the majority of overall births were attended by a midwife, although health officers were more common attendants in the low volume health centres (*P* < 0.001). Although most respondents (75.5%) had used the health centre previously to receive care for themselves, their children, or their spouses, the majority (70.1%) had not had a previous delivery at the same health centre. Previous health centre utilization differed significantly by health centre (*P* < 0.001 for each); respondents from the high-volume health centres were more likely to have previously used the health centre for any type of care and for delivery services.

### Facility characteristics

Facility data collected from on-site record books revealed that each health centre was differentially staffed and equipped (see Table 2). In the year preceding data collection, the low-volume health centres saw approximately one-third fewer births than the high-volume health centres in their respective regions, yet had more nurses and health officers as well as more BEmONC trained providers. The low-volume health centres also had fewer midwives on staff.

**Table 2.** Facility characteristics July 2013

Facility characteristics	Amhara		SNNP	
	High volume	Low volume	High volume	Low volume
Number of midwives	3	2	5	2
Number of nurses and health officers	11	12	14	16
Number trained on BEmONC	1	2	2	3
Number of delivery couches	2	1	4	2
Number of beds in the pre/post-labour ward	2	2	3	2
Number of annual deliveries	453	130	433	144

### D&A

#### Observed D&A

Of the 204 women who were sampled, 193 deliveries were directly observed and specific indicators of D&A were recorded (Table 3). Frequencies of several manifestations of D&A were high, with significant variation between health centres. The application of fundal pressure, an example of physical abuse, was recorded in as many as one-third of deliveries in ALV and one in five deliveries in SLV. Lack of consent for vaginal examination differed significantly between health centres, occurring in nearly all deliveries in the ALV and SHV, and less frequently in AHV and SLV.

**Table 3.** Observed disrespect and abuse

Type of disrespect and abuse observed	Total *N* = 193 *n* (%)	Frequency				Fisher’s exact *P*-value
		Amhara high volume *N* = 78 *n* (%)	Amhara low volume * N* = 15 *n* (%)	SNNP high volume *N* = 65 *n* (%)	SNNP low volume *N* = 35 *n* (%)	
Physical abuse						
Fundal pressure applied	22 (11.4)	1 (1.3)	5 (33.3)	9 (13.9)	7 (20.0)	<0.001*
Non-consented care						
Lack of consent for first vaginal examination	132 (68.4)	50 (64.1)	13 (86.7)	61 (93.9)	8 (22.9)	<0.001*
Non-confidential care						
Mother’s history taking findings shared when others could hear	64 (33.2)	37 (47.4)	7 (46.7)	3 (4.6)	17 (48.6)	<0.001*
Auditory privacy not respected during post-natal examination	41 (21.2)	29 (37.2)	3 (20.0)	2 (3.08)	7 (20.0)	<0.001*
Lack of privacy						
No partitions separating beds for first examination	109 (56.5)	24 (30.8)	15 (100.0)	48 (73.9)	22 (62.9)	<0.001*
Partitions do not give privacy in prenatal ward	53 (27.5)	37 (47.4)	0 (0)	12 (18.5)	4 (11.4)	<0.001*
Mother not covered during examination in prenatal ward	68 (35.2)	21 (26.9)	4 (26.7)	27 (41.5)	16 (45.7)	0.127
Mother not covered while being moved from prenatal ward to delivery room	42 (21.8)	15 (19.2)	3 (20.0)	20 (30.8)	4 (11.4)	0.149
Mother not covered during delivery	107 (55.4)	35 (44.9)	3 (20.0)	45 (69.2)	24 (68.6)	<0.001*
Partitions not closed during delivery	109 (56.5)	44 (56.4)	14 (93.3)	50 (76.9)	1 (2.9)	<0.001*
Mother not well covered after third stage of labour	60 (31.1)	20 (25.6)	3 (20.0)	20 (30.8)	17 (48.6)	0.086
No partitions/curtains between beds in post-natal ward	145 (75.1)	59 (75.6)	14 (93.3)	47 (72.3)	25 (71.4)	0.353
Mother’s physical privacy not respected during post-natal examination	40 (20.7)	28 (35.9)	3 (20.0)	0 (0)	9 (25.7)	<0.001*
Non-dignified care						
Mother not welcomed in a kind and gentle manner	24 (12.4)	10 (12.8)	6 (40.0)	8 (12.3)	0 (0)	0.002*
Provider did not introduce herself to mother (antenatal ward)	158 (81.9)	68 (87.2)	15 (100.0)	60 (92.3)	15 (42.9)	<0.001*
Use of non-dignified language during history taking	13 (6.7)	6 (7.7)	3 (20.0)	2 (3.1)	2 (5.7)	0.127
Delivery midwife did not introduce herself by name (if it was a provider mother had not yet met)	32 (16.6)	9 (11.5)	2 (13.3)	13 (20.0)	8 (22.9)	0.367
Delivering service provider did not congratulate mother after birth	62 (32.1)	12 (15.4)	10 (66.7)	32 (49.2)	8 (22.9)	<0.001*
Mother not cleaned after birth and third stage of labour	50 (25.9)	34 (43.6)	3 (20.0)	13 (20.0)	0 (0)	<0.001*
No pad provided to mother	88 (45.6)	59 (75.64)	2 (13.3)	19 (19.2)	8 (22.9)	<0.001*
Mother not allocated her own bed in post-natal ward	11 (5.7)	0 (0)	11 (73.3)	0 (0)	0 (0)	<0.001*
Bed in post-natal ward not clean	40 (20.7)	0 (0)	11 (73.3)	12 (18.5)	17 (48.6)	<0.001*
Mother not called by her name throughout interactions	65 (33.7)	43 (55.1)	5 (33.3)	16 (24.6)	1 (2.9)	<0.001*
Mother not asked about preferred birth position	162 (83.9)	70 (89.7)	15 (100.0)	46 (70.8)	31 (88.6)	0.005**
Mother not allowed to practice religious/cultural custom, if requested	10 (5.2)	8 (10.3)	1 (6.7)	0 (0)	1 (2.9)	0.022***

* *P* < 0.001;

** *P* < 0.01; ***P < 0.05.

Instances of non-confidential care were observed in up to half of all deliveries in three of four health centres, particularly during history taking in the admissions process. Lack of privacy—including lack/misuse of privacy curtains and women not covered during examinations and/or labour and delivery—were frequently recorded in all health centres, although specific manifestations differed significantly between health centres.

Finally, a wide range of non-dignified behaviours were observed, many with significantly variable distribution between health centres. Two thirds of women in ALV were not congratulated after giving birth compared to 15% in AHV and 23% in SLV. Despite having lower volume, women in ALV and SLV were more likely to be assigned to an unclean bed in the post-natal ward. Further, 83.9% of women observed were not asked about their preferred birth position; however, cultural customs were allowed during most births when requested by the woman.

#### Client reports of D&A

During exit interviews, one in five respondents (21.1%) reported experiencing some form of D&A during labour and delivery. Prevalence was significantly higher among health centres in the Amhara region (Table 4). The most commonly reported type of D&A was non-consented care (17.8%), with abdominal palpations and vaginal examinations both commonly reported examples (10.8% and 15.2% of respondents, respectfully, data not shown). Lack of privacy (15.2%) and non-confidential care (13.7%) were also highly reported. Every category of D&A was more frequently reported in the health centres in the Amhara region than those in SNNP region, and this difference was significant for overall D&A, non-consented care, lack of privacy, and non-confidential care (*P* < 0.001 for each).

**Table 4.** Reported experiences of disrespect and abuse by facility

Type of D&A	Total	Amhara		SNNP		*P*-value
		High volume *N* = 77	Low volume *N* = 21	High volume *N* = 71	Low volume *N* = 35	
	*n* (%)	*n* (%)	*n* (%)	*n* (%)	*n* (%)	
Any form of D&A	43 (21.1)	30 (39.0)	8 (38.1)	4 (5.6)	1 (2.9)	<0.001*
Physical abuse	1 (0.5)	1 (1.3)	0 (0)	0 (0)	0 (0)	1.000
Non-consented care	36 (17.8)	26 (33.8)	7 (33.3)	3 (4.2)	0 (0)	<0.001*
Lack of privacy	31 (15.2)	25 (32.5)	4 (19.1)	1 (1.4)	1 (2.9)	<0.001*
Non-confidential care	28 (13.7)	22 (28.6)	5 (23.8)	1 (1.4)	0 (0)	<0.001*
Non-dignified care	2 (1.0)	2 (2.6)	0 (0)	0 (0)	0 (0)	0.736
Abandonment	5 (2.5)	4 (5.2)	1 (4.8)	0 (0)	0 (0)	0.092
Detention	0 (0)	0 (0)	0 (0)	0 (0)	0 (0)	NA

* *P* < 0.001.

#### Unadjusted associations with reporting of D&A

In bivariate analyses (Table 5, Unadjusted), religious affiliation was highly associated with reporting of D&A, with Muslim women being significantly less likely to report experiencing D&A than Christian women [odds ratio (OR) = 0.16, 95% CI 0.05, 0.47] (data not shown). Women from urban *kebeles* (villages) were 2.5 times as likely to report experiencing D&A than those from rural areas (OR = 2.48, 95% CI 1.25, 4.92). Women who experienced any complications or had an infant who experienced complications were eight times more likely to report any D&A than women who did not (OR = 7.98, 95% CI 3.70, 17.22). Compared to women who delivered during the day on a weekday, women who delivered on the weekend were 83% less likely to report any D&A (OR = 0.17, 95% CI 0.05, 0.063). Women who delivered at night were also less likely to report D&A than those who delivered during the day, although the difference was not significant (OR = 0.69, 95% CI 0.33, 1.45). Finally, women who had not previously delivered at the study health centre were 3.2 times more likely to report experiencing any form of D&A than women who had delivered previously at the health centre (OR = 3.2, 95% CI 1.27, 8.05). However, the cadre of birth attendant at the time of delivery was not significantly associated with reporting of D&A.

**Table 5.** Odds ratios of respondents experiencing any disrespect and abuse during childbirth, crude and adjusted analysis

Characteristics	Unadjusted		Adjusted	
	OR	(95% CI)	OR	(95% CI)
Age group				
16–24	1.00		1.00	
25–34	1.17	(0.56–2.46)	1.98	(0.59–6.66)
35–45	1.33	(0.45–3.95)	0.45	(0.08–2.44)
Education				
None	1.00		1.00	
Primary	1.19	(0.55–2.56)	3.11	(0.89–10.85)
Secondary+	1.32	(0.55–3.21)	2.94	(0.55–15.66)
Marital status				
Other	1.00		1.00	
Married	0.65	(0.19–2.17)	0.84	(0.16–4.39)
Occupation				
Homemaker	1.00		1.00	
Farming	1.32	(0.60–2.90)	1.91	(0.37–9.8)
Other	1.05	(0.44–2.50)	0.31	(0.06–1.48)
Wealth tercile				
Poorest	1.00		1.00	
Medium	1.41	(0.62–3.19)	3.20	(0.71–14.39)
Least poor	1.00	(0.13–0.44)	3.37	(0.54–20.92)
Place of residence				
Rural	1.00		1.00	
Urban+	2.48*	(1.25–4.92)	1.64	(0.29–9.32)
Travel time to reach the facility				
<1 h	1.00		1.00	
1–2 h	0.59	(0.25–1.39)	0.23*	(0.06–0.89)
>2 h	0.85	(0.31–2.28)	0.70	(0.17–3.00)
Referred to the facility				
Referred	1.00		1.00	
Came directly	0.38	(0.10–1.40)	0.40	(0.04–3.62)
Any complications				
No	1.00		1.00	
Yes	7.98*	(3.70–17.22)	15.51*	(4.38–54.94)
Delivery time				
Weekdays day	1.00		1.00	
Weekdays night	0.69	(0.33–1.45)	0.53	(0.15–1.80)
Weekend	0.17*	(0.05–0.63)	0.05*	(0.01–0.32)
Birth attendant				
Health officer	1.00		1.00	
Nurse	2.37	(0.84–6.66)	2.11	(0.35–12.74)
Midwife	1.39	(0.58–3.34)	1.21	(0.29–5.14)
Number of ANC visits				
0–2	1.00		1.00	
3–4	0.66	(0.27–1.61)	0.55	(0.13–2.27)
Previously delivered at the facility				
Yes	1.00		1.00	
No	3.20*	(1.27–8.05)	1.66	(0.47–5.8)
Previously used the facility				
Yes	1.00		1.00	
No	0.92	(0.42–2.03)	0.63	(0.19–2.13)

* *P* < 0.05.

#### Adjusted associations with reporting of D&A

A facility-level fixed effects logistic regression model was estimated to assess the associations between reported D&A and client characteristics (Table 5, Adjusted). Experience of maternal or neonatal complications and delivery time remained significant predictors of reporting of D&A, as did the specific health facility. Religious affiliation was collinear with the facility type indicator (i.e. Muslims were only in SHV), and thus not included in the model. Women who experienced any complications or whose newborn experienced any complications were 15.51 times more likely to report any D&A than women who did not (OR = 15.51, 95% CI 4.38, 54.94) when controlling for the facility and client characteristics. Women who delivered on the weekend were 95% less likely than women who gave birth during the day on a weekday to report any D&A (OR = 0.05, 95% CI 0.01, 0.32).

## Discussion

The objective of our study was to understand the manifestations of D&A in four rural health centres for the purposes of informing community-led interventions. To ensure a comprehensive approach, we employed tools that captured two perspectives: observed D&A as recorded by clinicians trained to observe client-provider interactions, and experienced D&A as reported by women who gave birth in the health centres. During the client-provider interactions, non-dignified care was observed most often (83.9%), although types varied significantly between health centres. Of the women interviewed post-partum, 21.1% reported experiencing any type of D&A, and birth complications increased the odds of reporting D&A by nearly eight-times in the unadjusted analyses, which doubled when adjusted for socio-demographic and index birth characteristics.

The significant variance in frequencies and types of observed D&A between health centres reinforce the theory that efforts to address D&A require a localized effort ‘where women live and labour’ (Freedman and Kruk 2014). However, frequencies of some manifestations were high across all health centres, including structural deficiencies (i.e. no partitions separating beds for first examination) and breakdowns in preferred client-provider interactions (i.e. mother not asked about preferred birth position), indicating there is a role for larger systemic support for improved infrastructure as well as increased accountability for standards of care. During exit interviews, more than one in five women reported experiencing some type of D&A while giving birth, comparable to prevalence measures reported by colleagues using similar tools **(**Kruk *et al.* 2014; Abuya et al. 2015a; Ratcliffe et al. 2016a; Sando *et al.* 2016). Overall D&A varied significantly by health centre, and was more prevalent in Amhara region than SNNP region.

In using multiple methods to measure D&A, several interesting patterns emerged. The frequency of D&A was higher at direct observation than reported by women during the exit interview, which was consistent across all health facilities. The differences in frequency of observed D&A between regions was less straightforward, and no clear pattern emerged as to which region had more D&A observed. For example, the single non-consent variable (lack of consent for first vaginal information) was observed in 68.4% of women but non-consented care was reported by only 17.8% of women. This discrepancy varied significantly by facility: Amhara high volume 64.1% observed vs 33.8% reported; Amhara low volume 86.7% observed vs 33.3% reported; SNNP high volume 93.9% observed vs 4.2% reported; and SNNP low volume 22.9% observed vs 0% reported. These inconstant data underline the need to tailor D&A interventions to facilities and their surrounding communities, while bearing in mind that individual birth characteristics may render a woman more at risk for experiencing D&A.

The discrepancies in observed vs experienced D&A are among the most discussed in the field (Rosen *et al.* 2015; Sando et al. 2017). Freedman *et al.* (2014) note that D&A is not a single definition, but a confluence of experiences, drivers and external factors; these include normalization of behaviour and circumstances by both woman and provider, mistreatment due to failing infrastructure, and deviations from professional norms and standards. One could presume that using data collectors with a background as health care providers may have introduced bias towards underreporting of D&A; yet, D&A prevalence was higher when scored by providers than reported by women. The larger difference between observed and reported D&A in SNNP region suggests that women may have normalized D&A to a greater degree than women from the Amhara region.

Further examination of predictors shows that most client characteristics in the unadjusted models were not significantly associated with reporting of D&A. For example, women’s age and education were not significantly associated with reporting of D&A in the unadjusted or adjusted models, which was consistent with findings from Tanzania, Kenya and Nigeria (Kruk *et al.* 2014; Okafor *et al.* 2014; Abuya et al. 2015a). Among index birth characteristics, reported birth complications were most strongly associated with increased odds for reporting D&A, and the magnitude nearly doubled when adjusted. It could be argued that more complicated deliveries are more stressful for health care providers, which lowers the quality of services provided. Alternatively, we could hypothesize that women who have complicated pregnancies are more prone to perceive the way they are treated as disrespectful. Further, the analyses were based on complications that women *reported*, as opposed to complications documented in a medical record, and it could be that those who experienced D&A were more likely to report complications. Although the directionality of the association cannot be determined concretely, the association of birth complications and D&A merits further investigation.

Of particular note were the unexpected associations with D&A. Although midwifery care has been identified as a contributing factor to higher quality, respectful care (Renfrew *et al.* 2014), and the majority of births in our study were attended by midwives, there was no significant association between midwife attendance at birth and reporting of D&A. Weekend delivery, often considered a risk factor for obstetric complications (Janakiraman *et al.* 2011), was protective across models, and the association was even stronger when adjusted for socio-demographic and index birth characteristics. Childbirth was the only maternal health service offered on weekends at the study health centres, which may have contributed to lower caseload per provider. Patient flow has been suggested as an environmental factor that may contribute to D&A, and that high patient volume or low staff count would be associated with high D&A because staff are overworked, possibly burned out, and/or not able to perform tasks beyond what is considered necessary to ensure maternal and newborn survival (D’Oliveira *et al.* 2002; Bowser and Hill 2010). Thus, lower reports of D&A may reflect more personalized attention given to women compared to women who give birth during the weekday. These data are important considerations when recommending that health systems examine supply-side factors that contribute to maternal death and morbidity (Knight *et al.* 2013). This will be of particular importance to Ethiopia, where some regions have reported a 10-fold increase in facility-based delivery over a 7 year period within the past decade (The Last Ten Kilometers Project 2015), raising concerns about whether facilities are adequately equipped and staffed to accommodate this increased patient flow.

Finally, although infrastructure challenges may have contributed to D&A observed during client-provider interactions, D&A has also been documented in health care facilities that are well-staffed and stocked (Jewkes *et al.* 1998). Our observations showed that providers were sometimes active perpetrators of D&A. Rarely were women asked about their preferred birth position, nor did providers routinely obtain consent for vaginal examinations, introduce themselves to women or congratulate women after giving birth. These instances provide an opportunity to improve specific patient-centred practices during routine training at the health centres.

### Limitations

The study had several limitations. First, as the study sites were selected based on a set of criteria, but were not representative of all clinics in these regions, the results of the study are not generalizable to other health facilities within Ethiopia. Additionally, the health centres were part of a larger initiative for community-led quality improvement; therefore, it is possible that the study sites were more attuned to professional standards and were more attentive to required facility maintenance than other facilities in the region. Although all women giving birth at the facilities were invited to participate in the study, the sample is inherently biased, as the vast majority of women in Ethiopia do not give birth in health facilities (Central Statistical Agency 2014). Women who opted to give birth in facilities may have been more or less likely to report D&A than other women in Ethiopia. For example, more than half of women interviewed reported living less than 1-h travel distance from the health centre; it is possible that the sample were more likely to know their provider—whether through previous interactions with the health system or social connections—and perhaps less likely to report D&A.

Second, the sample size was powered to estimate overall D&A prevalence and not powered to detect variability of D&A by the independent variables. As such, some of non-significant effects of client characteristics (e.g. education and wealth) or index birth characteristics (e.g. number of ANC visits) that showed strong associations could be due to lack of power of the sample. Also, limiting the sample size to four facilities prevented the ability to examine associations between facility characteristics and D&A.

Third, exit interviews immediately post-partum are a debated method of obtaining data on D&A (Glick 2009; Kruk *et al.* 2014). Three to 6 h post-partum may be too soon to interview women, as they may be exhausted, not wanting to engage, more focused on the status of their infant, and/or have not yet reflected on the birthing experience. Other studies have complemented exit interviews with community interviews conducted four to ten weeks post-delivery to compare reported D&A (Kruk *et al.* 2014; Sando *et al.* 2016); unfortunately, the study budget did not allow for this method. Fourth, although every effort was made to assure women that data collectors were not affiliated with the health centre and that their responses were anonymous, interviewing women on facility grounds may have increased the chance of courtesy bias. It is also possible that women felt fear of retaliation from health care providers and staff should they report negative experiences while at the health centre.

Finally, it is highly complex to measure D&A through observation. Despite recent progress, D&A has yet to be universally defined or operationalized (Freedman *et al.* 2014; Bohren *et al.* 2015; Vogel *et al.* 2015a,b) and some instances of D&A are entirely subjective, and may not be captured by a third-party observer. While the data collectors came to consensus on how to observe each item during training, we are limited in that the operationalization was not formally documented. Further, the presence of the observer him/herself may influence the prevalence of some D&A behaviours. However, if behaviours are normalized enough within the clinical setting, then it is less likely to affect practice. The tool employed by this study was designed to capture specific instances of D&A, but did not ask data collectors to comprehensively document *any* instances of D&A. For example, observers noted incidence of fundal pressure as physical abuse, but were not prompted throughout the observation to document *all* instances of physical abuse, such as pinching and slapping, which have been observed and reported in other settings (Jewkes *et al.* 1998; Okafor *et al.* 2014; Abuya et al. 2015b; Ratcliffe *et al.* 2016b). Therefore, the information collected through this tool is somewhat incomplete and does not yield a comprehensive observed prevalence of D&A. Because of this limitation, direct comparisons of overall observed prevalence to overall prevalence from women’s reports are not possible. We recommend that those intending to measure observed D&A include a prompt to document all instances of D&A during client-provider interactions. While the maternal health field is debating what method is the ‘best’ or ‘most accurate’ to define, describe, and report D&A, studies should seek to test different approaches and methodologies, given previous research and practical constraints, and report on them and their findings to allow for comparison between methods to build the evidence base for the field.

## Conclusion

D&A is a violation of human rights and a threat to achieving good maternal health outcomes. To date, this is one of the first studies to quantify the prevalence of D&A at the PHCU level in Ethiopia, the frontline of facility-based childbirth in the national health care system. As access to facility-based childbirth increases across the country, the data presented here provide evidence of a much-needed shift in priorities towards improving the quality of care, including respect and dignity during childbirth. Future efforts should include nationwide policies and initiatives in improving facility infrastructure and enforcing accountability of professional standards, as well as local, facility-specific interventions to improve comprehensive quality of care that benefits women and their babies, health care providers, and the community at large.

## Footnotes

1 Woredas are administrative areas with an average population of about 100 000 people.

2 Staffed with two female health extension workers (HEWs), the health posts provide community-based basic promotive, preventive and curative health services to a *kebele* (i.e. villages) with about 5000 people.

3 Maternal and fetal complications included extreme pain, high blood pressure/seizures/blurred vision/severe headache, swelling in hands/feet, fetal distress; or high birthweight, prolonged labour (more than 12 h), post-partum haemorrhage, infection (fever). Newborn complications included respiratory distress, signs of infection, difficulty feeding and jaundice.

4 The wealth index score was constructed for each of the respondents with the principal component analysis of the household possessions (electricity, watch, radio, television, mobile phone, telephone, refrigerator, table, chair, bed, electric stove and kerosene lamp), and household characteristics (main material of roof, type of latrine, water source). The households were ranked according to the wealth score and then divided into three terciles indicating poorest, medium and least poor households (Filmer and Pritchett, 2001).
